# FNDC5 inhibits autophagy of bone marrow mesenchymal stem cells and promotes their survival after transplantation by downregulating Sp1

**DOI:** 10.1038/s41420-023-01634-4

**Published:** 2023-09-06

**Authors:** Huan Wei, Shuaiye Liu, Tingting Wang, Yanping Li, Kangmei Liu, Qunying Guo, Ling Li

**Affiliations:** 1 Department of Neurology, The First Affiliated Hospital, Sun Yat-sen University; Guangdong Provincial Key Laboratory of Diagnosis and Treatment of Major Neurological Diseases, National Key Clinical Department and Key Discipline of Neurology, Guangzhou, China; 2 Department of Neurology, Yan’an Hospital of Kunming City; The Affiliated Yan’an Hospital of Kunming Medical University, Kunming, China; 3 Department of Cardiovascular Disease, The Seventh Affiliated Hospital, Sun Yat-sen University, Shenzhen, China; 4 Department of Geriatrics, Yan’an Hospital of Kunming City; The Affiliated Yan’an Hospital of Kunming Medical University, Kunming, China; 5 Department of Nephrology, The First Affiliated Hospital, Sun Yat-sen University, Guangzhou, China

**Keywords:** Stroke, Stem-cell research, Mesenchymal stem cells

## Abstract

Regenerative therapy based on mesenchymal stem cells (MSCs) has great promise to achieve functional recovery in cerebral infarction patients. However, the survival rate of transplanted MSCs is extremely low because of destructive autophagy caused by the harsh ischemic microenvironment in cerebral infarct tissue. The mechanism by which fibronectin type III domain protein 5 (FNDC5) regulates autophagy of transplanted bone marrow-MSCs (BMSCs) following ischemic injury needs to be elucidated. In this study, we confirmed that FNDC5 promotes the survival of transplanted BMSCs in a rat cerebral infarction model. Furthermore, bioinformatic analysis and verification experiments revealed the transcription factor, Sp1, to be a key mediator of autophagy regulation by FNDC5. FNDC5 significantly inhibited BMSC autophagy by down-regulating Sp1 and the autophagy-related Sp1-target gene, *ULK2*. Transplanted BMSCs overexpressing FNDC5 (BMSCs-OE-FNDC5) promoted neurovascular proliferation and alleviated ischemic brain injury in cerebral infarct model rats. However, the increased survival and enhanced neuroprotective effect of transplanted BMSCs-OE-FNDC5 were reversed by simultaneous overexpression of Sp1. Our data indicate a role for FNDC5 in BMSC survival and reveal a novel mechanism of transcription regulation through Sp1 for the autophagy-related gene *ULK2*. Modulation of FNDC5 may promote survival capacity and improve the therapeutic effect of BMSCs in various tissues following ischemia.

## Introduction

Stroke is a leading cause of adult disability [1] and the Chinese population ranks first in the world for the lifetime risk of stroke [2]. Among the existing 28.76 million stroke cases in China, 84% are cerebral infarction patients [3]. Unfortunately, various types of therapy, including thrombolysis and mechanical thrombectomy, are ineffective at continuously improving the overall disease prognosis [4]. Regenerative therapy based on mesenchymal stem cells (MSCs) has great promise for achieving functional recovery in cerebral infarction patients [5, 6]. By affecting ischemia pathophysiology at multiple targets, MSCs are considered to be “seed cells”, that are suitable for cell transplantation therapy after ischemic brain injury [7, 8]. However, the survival rate of transplanted MSCs is extremely low because of the severe microenvironment following ischemia, which greatly limits the therapeutic efficacy of MSCs for cerebral infarction [9].

The protective efficacy of MSCs is positively correlated with the number of transplanted cells that survive [10]. The low survival rate of transplanted MSCs is mainly attributed to extreme autophagy, apoptosis, and necrosis caused by persistent ischemia and hypoxia [11]. Among diverse types of cell death, autophagy is considered to both improve and hinder the survival, engraftment, and paracrine activities of MSCs in vivo [12, 13]. On the one hand, this highly conserved cell process provides protective effects against hypoxia or ischemia-induced injury by eliminating macromolecules that are no longer needed and by supplying extra energy [14, 15]. On the other hand, if the hypoxia or ischemic event is severe or prolonged, the autophagic process may be continuously activated, leading to enhanced MSCs death [16, 17]. Moreover, the interconnected nature of cell death pathways means that crosstalk between autophagy and apoptosis is likely to be consequential but this remains under investigation [18]. The detailed role of autophagy in hypoxia-induced apoptosis of MSCs has not been fully elucidated [16, 18]. Therefore, the highly networked and precise molecular mechanisms involved in the regulation of autophagy-related cell death require further exploration.

The glycosylated transmembrane precursor protein, Fibronectin type III domain protein 5 (FNDC5), and its extracellular cleavage product of 112 amino acids, named Irisin, have been implicated in numerous processes, including energy metabolism, oxidative stress, anti-aging, and synapse rebuilding [19, 20], making it a promising target for modulating autophagy [21]. Exercise-induced FNDC5/Irisin can protect nucleus pulposus cells against senescence by modulating autophagy [22] and we have previously shown that overexpression of FNDC5 (OE-FNDC5) can ameliorate autophagy and promote cell proliferation in bone marrow MSCs (BMSCs) upon hypoxia and serum deprivation (H/SD) [23]. However, the molecular pathways by which FNDC5 modulates BMSCs autophagy are undetermined and the protective effect of FNDC5 after BMSC transplantation for treatment of cerebral infarction needs further elucidation.

Here, we clarified the molecular mechanism by which FNDC5 regulates autophagy of BMSCs and affects their survival under H/SD conditions. Furthermore, we investigated the effects of FNDC5 on BMSC transplantation and neurological rehabilitation in middle cerebral artery occlusion (MCAO) rats and verified the hypothesis that FNDC5 is a key mediator of the beneficial effects of physical exercise after MSCs therapy in cerebral infarction models. FNDC5 is, therefore, a potential target for therapeutic intervention in treating cerebral infarction.

## Results

### Enhanced autophagy exacerbates cell death under conditions of hypoxia and serum deprivation

**Fig. 1:**
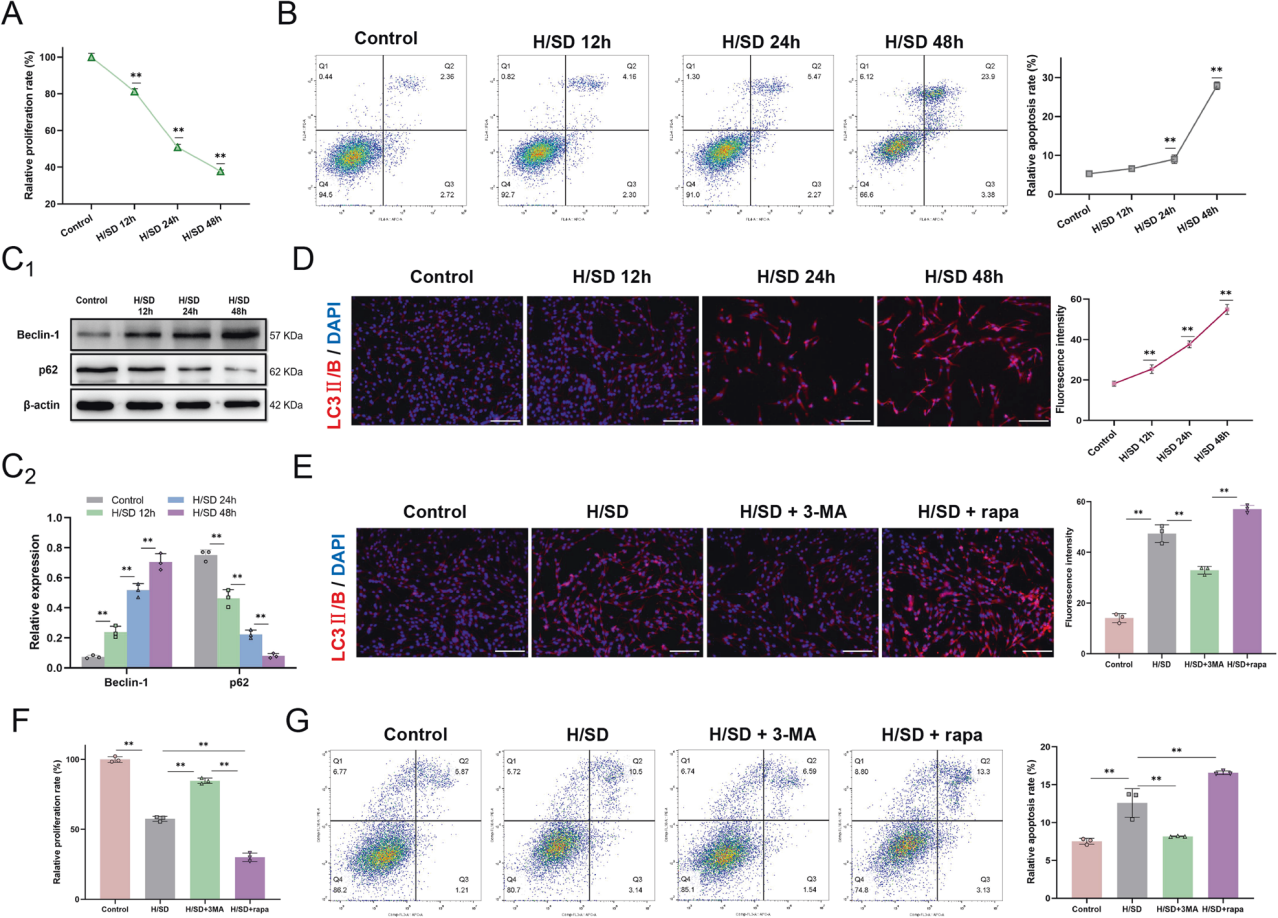
Effects of H/SD on BMSC autophagy and viability. **A** CCK-8 assay detection of cell proliferation after H/SD treatment. **B** Flow cytometry detection of apoptosis upon H/SD treatment. **C** Western blot analysis of autophagy representative proteins, Beclin 1 and p62. **D** Immunofluorescence of LC3 II/B in BMSCs at different times after injury. **E** Immunofluorescence of LC3 II/B after H/SD and autophagy inhibitor (3-MA, 5 mM) or autophagy inducer (rapa, 1.0 μg/L) treatment. **F** CCK-8 assay detection of cell proliferation after autophagy inhibition or promotion. **G** Flow cytometry detection of BMSC apoptosis after autophagy inhibition or promotion. Data are presented as the mean ± SD, *p < 0.05, **p < 0.01, *n* = 3, Scale bar = 100 μm. H/SD hypoxia/serum deprivation, LC3 II/B Microtubule-associated protein 1 light chain 3 II/B, DAPI 4'6-diamidino-2-phenylindole, 3-MA 3-methyl-adenine, rapa rapamycin.

To explore the effect of severe hypoxia and ischemia on cell death, we subjected BMSCs to H/SD for 12, 24, and 48 h. CCK8 assays showed that cell proliferation decreased gradually with extended injury time (Fig. 1A), and flow cytometry showed that BMSC apoptosis significantly increased with prolonged injury (Fig. 1B). In addition, for two representative autophagy proteins, levels gradually increased for Beclin 1 and decreased for p62 (Fig. 1C). Accordingly, the fluorescence intensity of the autophagy marker, LC3 II/B, was enhanced with persistent H/SD (Fig. 1D). These results indicated that sustained H/SD enhanced BMSC autophagy and exacerbated cell death.

The effect of autophagy on the apoptosis and proliferation of BMSCs was further explored using an autophagy inhibitor, 3-methyl-adenine (3-MA), and an autophagy activator, rapamycin (rapa). Immunofluorescence analysis indicated that LC3 II/B levels were decreased, and autophagy was inhibited in the 3-MA (5 mmol/L)-treated group under H/SD for 24 h. In contrast, addition of rapa (1.0 μg/L) promoted autophagy and increased the level of LC3 II/B (Fig. 1E). Concomitant with the enhancement of autophagy (in the rapa group), apoptosis increased, and proliferation decreased under H/SD. However, inhibition of autophagy (in the 3-MA group) promoted BMSC viability and reduced apoptosis (Fig. 1F, G). Together, these results confirmed that inhibition of autophagy alleviated apoptosis and improved the survival of BMSCs under persistent and severe hypoxia and nutrient deprivation.

### Overexpression of FNDC5 inhibits autophagy in BMSCs under H/SD

**Fig. 2:**
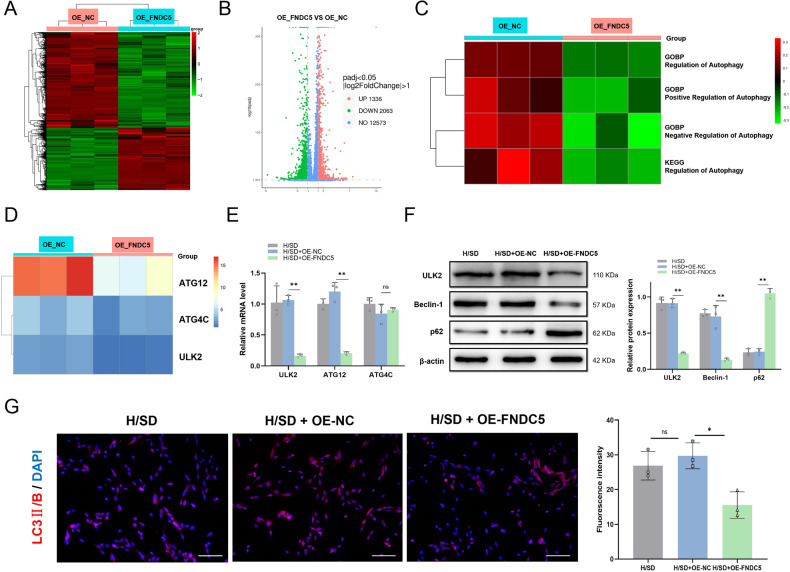
Effects of OE-FNDC5 on autophagy of BMSCs subject to H/SD. **A** Heatmap of differentially expressed genes determined by RNA-seq analysis of BMSCs transfected with lentivirus-FNDC5 (OE-FNDC5) or vehicle control (OE-NC) and subjected to H/SD for 24 h. **B** Volcano plot of RNA-seq of BMSCs transfected with OE-FNDC5 lentivirus or vehicle and subjected to H/SD for 24 h. **C** GO and KEGG cluster analysis of autophagy-related pathways that were affected by OE-FNDC5. **D** Top three autophagy-related genes (*ATG12*, *ATG4C*, and *ULK2*) that were significantly inhibited by OE-FNDC5. **E** qRT-PCR of the top three autophagy-related genes. **F** Western blotting and quantification of autophagy-related protein, ULK2, and autophagy representative proteins, Beclin 1 and p62, in BMSCs. **G** Immunofluorescence of LC3 II/B in BMSCs subjected to H/SD and OE-FNDC5 treatment. Data are presented as the mean ± SD, *p < 0.05, **p < 0.01, *n* = 3, Scale bar = 100 μm. OE-FNDC5 overexpression of FNDC5, OE-NC overexpression negative control, ATG autophagy related gene, GOBP Gene Ontology biological process, KEGG Kyoto Encyclopedia of Genes and Genomes.

We previously demonstrated the effect of FNDC5 overexpression in BMSCs under H/SD on autophagy and promotion of cell survival [23]. In this study, we continued to explore the molecular mechanisms by which FNDC5 regulates autophagy in BMSCs. High-throughput RNA sequencing (RNA-seq) was performed on BMSCs transfected with lentivirus overexpressing FNDC5 (BMSCs-OE-FNDC5) and on negative control BMSCs (BMSCs-OE-NC) that had been subjected to H/SD for 24 h. OE-FNDC5 caused significant down-regulation of 2063 genes (Fig. 2A, B). Gene Ontology (GO) and Kyoto Encyclopedia of Genes and Genomes (KEGG) analysis identified the biological process “regulation of autophagy” as significantly enriched and downregulated by OE-FNDC5 (Fig. 2C).

We focused on the most significantly downregulated autophagy-related genes, *ATG12*, *ATG4C,* and *ULK2* (Fig. 2D). We performed qPCR and western blot analyses to validate their expression in BMSCs. The mRNA levels of *ATG12* and *ULK2* were significantly decreased by OE-FNDC5 (Fig. 2E). OE-FNDC5 dramatically decreased the protein level of ULK2 and the autophagy representative protein, Beclin 1, but increased the level of p62 (Fig. 2F). Similarly, OE-FNDC5 reduced LC3 II/B fluorescence in hypoxic BMSCs (Fig. 2G), which was consistent with our previous experimental results [23].

### FNDC5 downregulates transcription factor Sp1 in BMSCs under H/SD

**Fig. 3:**
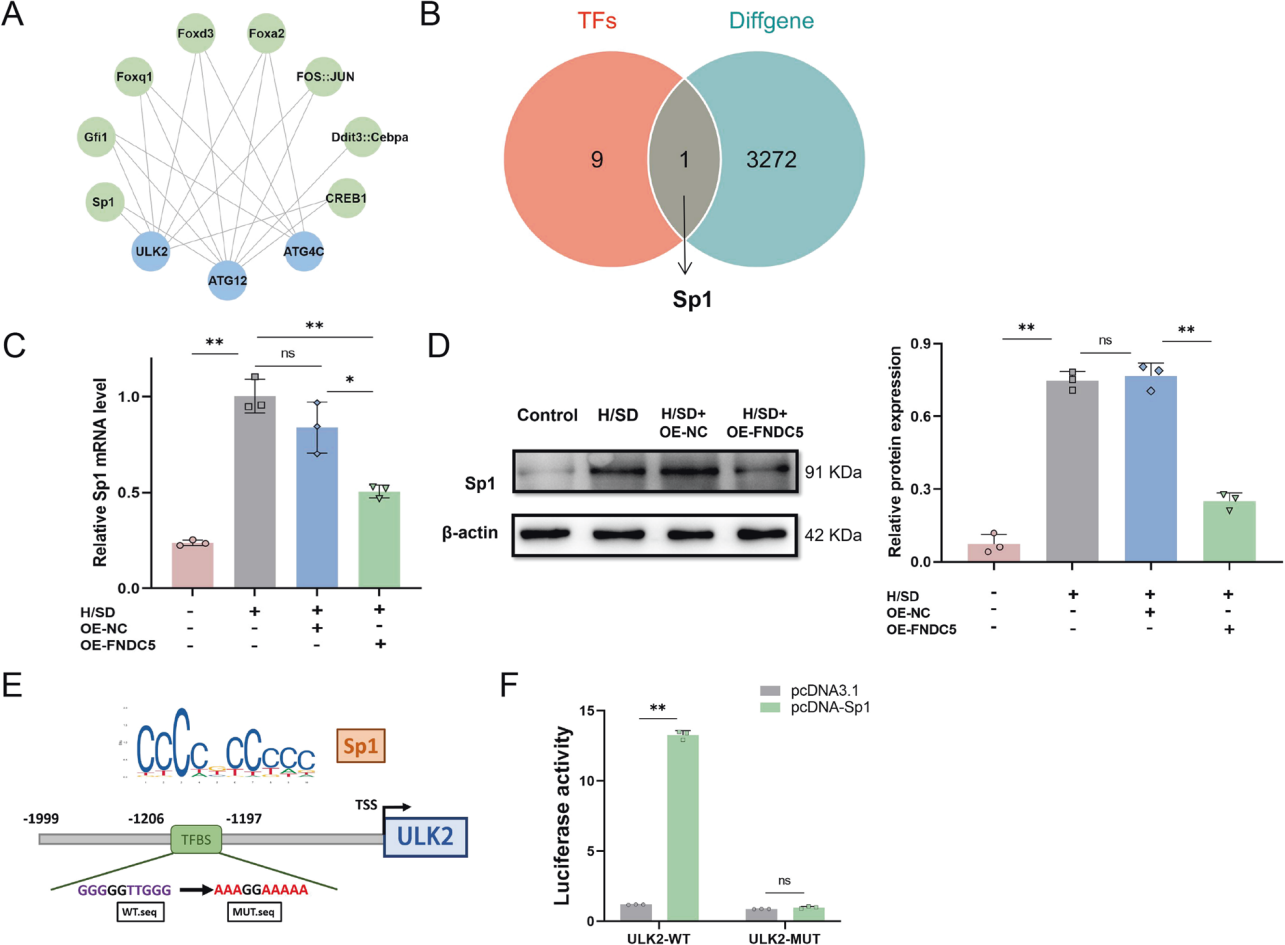
Sp1 is the key transcription factor regulating ULK2 in BMSCs-OE-FNDC5 subject to H/SD. **A** Protein-protein interaction network of three autophagy-related genes and their transcription factors identified from the JASPAR database. **B** Venn diagram of the 10 screened transcription factors and the genes that were differentially expressed between BMSCs-OE-FNDC5 vs BMSCs-OE-NC determined by RNA-seq. **C** qRT-PCR of *Sp1* gene expression in BMSCs-OE-FNDC5 and BMSCs-OE-NC subjected to H/SD. **D** Western blots and quantification of Sp1 protein in BMSCs treated with OE-FNDC5 or vehicle control. **E** Predicted positions of transcription factor binding sites in the full-length *ULK2* promoter and the mutant sequence. **F** Dual-luciferase reporter gene assay of transcription factor, Sp1, and its target gene, *ULK2*. Data are presented as the mean ± SD, *p < 0.05, **p < 0.01, *n* = 3, Scale bar = 100 μm. TFs transcription factors, Diffgene differentially expressed genes, TFBS transcription factor binding sites, TSS transcription start site, WT wild-type, MUT mutant.

We then predicted transcription factors that regulate the three differentially expressed autophagy-related genes, *ATG12*, *ATG4C,* and *ULK2*. We obtained their FASTA sequences from the JASPAR database and produced a protein-protein interaction diagram that shows the relationships among the autophagy-related genes and their potential transcription factors (Fig. 3A). Ten transcription factors were acquired and intersected with differentially expressed genes (DEGs) from the original RNA-seq results. This analysis identified the transcription factor, Sp1 (Fig. 3B). qPCR and western blotting results showed that the expression of Sp1 was enhanced under H/SD but inhibited by OE-FNDC5 (Fig. 3C, D).

To further clarify the direct interaction between Sp1 and its target gene, *ULK2*, we performed luciferase assays to confirm the transcription factor binding sites. First, we generated a schematic diagram of the DNA binding domain (Fig. 3E) and identified the most probable transcription factor binding site sequence (−1206 to −1197 bp) in the *ULK2* promoter by mining the JASPA database. Plasmid vectors containing mutant and wild-type versions of this sequence were constructed and transfected into 293 T cells. The luciferase activity in the ULK2-MUT group was significantly decreased, indicating direct regulation of *ULK2* transcription by Sp1 (Fig. 3F). Collectively, bioinformatic analysis and in vitro experimental verification confirmed that OE-FNDC5 can inhibit autophagy of BMSCs under H/SD by down-regulating the transcription factor, Sp1, and its autophagy target gene, *ULK2*.

### Overexpression of Sp1 promotes autophagy and reduces the viability of BMSCs under H/SD

**Fig. 4:**
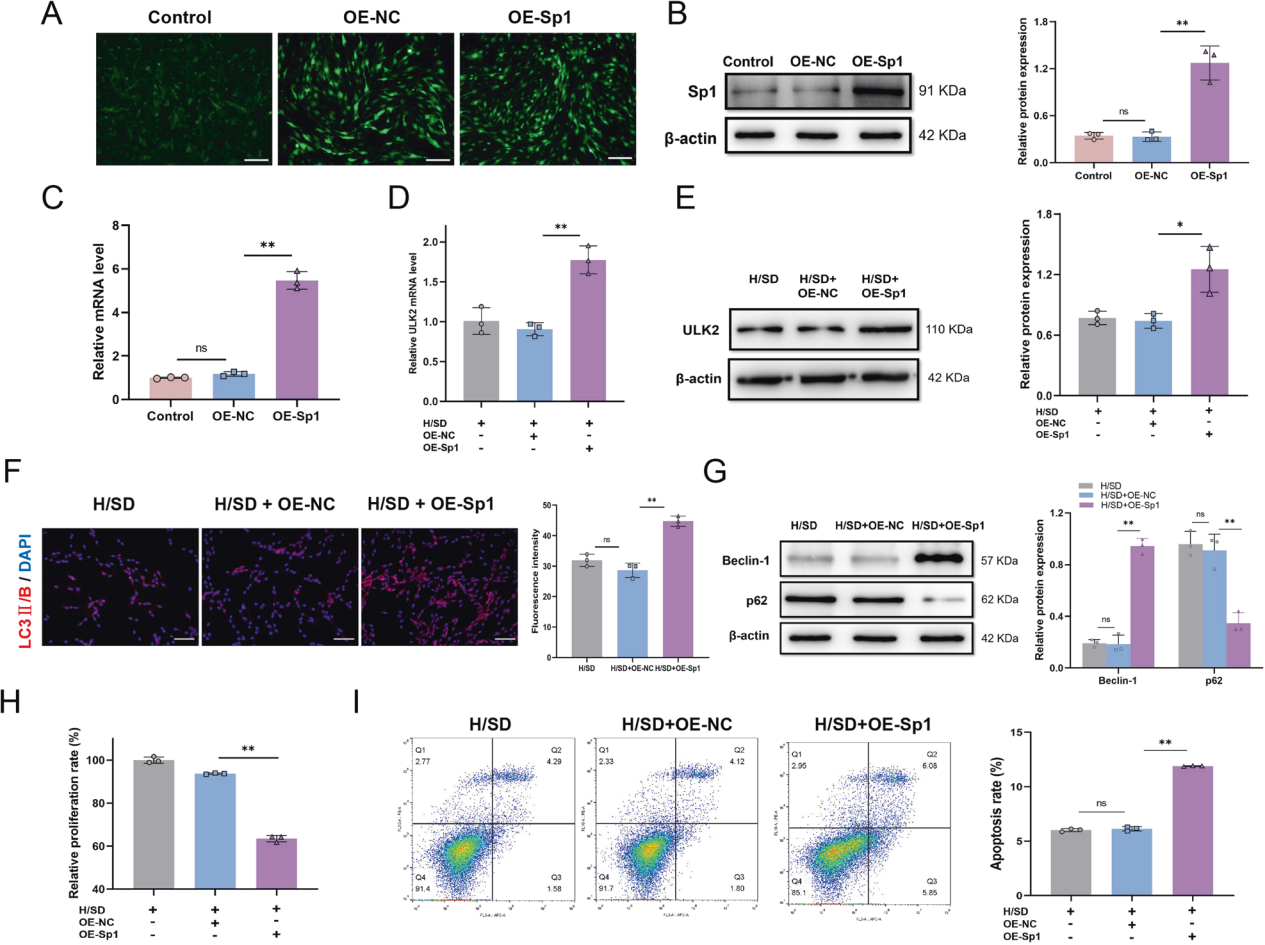
Effects of OE-Sp1 on autophagy and viability of BMSCs subject to H/SD. **A**–**C** Validation of Sp1 overexpression in BMSCs. **A** Immunofluorescence confirmed the transfection efficiency of OE-Sp1 lentivirus, which was GFP-labeled. **B** Western blotting confirmed Sp1 protein overexpression. **C** qRT-PCR detected overexpression of Sp1 mRNA in transfected BMSCs. **D** qRT-PCR detection of *ULK2* mRNA levels in BMSCs-OE-Sp1 and BMSCs-OE-NC subject to H/SD. **E** Western blotting of ULK2 protein in BMSCs treated with OE-Sp1 lentivirus or vehicle control. **F** Immunofluorescence of LC3 II/B in BMSCs after OE-Sp1 lentivirus or vector control transfection. **G** Western blotting of autophagy representative proteins, Beclin 1 and p62, in BMSCs. **H** CCK-8 assay detection of cell proliferation after OE-Sp1 or OE-NC treatment and H/SD. **I** Flow cytometry detection of BMSC apoptosis after OE-Sp1 lentivirus or vector control transfection. Data are presented as the mean ± SD, *p < 0.05, **p < 0.01, *n* = 3, Scale bar = 100 μm. OE-Sp1 overexpression of Sp1, OE-NC overexpression negative control.

To further explore the effect of Sp1 on BMSC autophagy, we constructed BMSC lines overexpressing Sp1 (BMSCs-OE-Sp1) by lentivirus transfection. Representative immunofluorescence images showed that BMSCs-OE-Sp1 or BMSCs-OE-NC, which were transfected with GFP-tagged lentivirus, had stable viability (Fig. 4A). Western blotting (Fig. 4B) and qRT-PCR (Fig. 4C) confirmed overexpression of Sp1 in BMSCs-OE-Sp1. Next, we further explored the effect of Sp-1 overexpression (OE-Sp1) on the autophagy target protein, ULK2, in BMSCs under H/SD. OE-Sp1 up-regulated both mRNA and protein levels of ULK2 compared with OE-NC cells (Fig. 4D, E). Immunofluorescence staining for LC3 II/B showed that OE-Sp1 increased the level of LC3 II/B in BMSCs under H/SD (Fig. 4F). Moreover, western blotting of another two autophagic marker proteins revealed increased Beclin 1 and decreased p62 levels in response to OE-Sp1 (Fig. 4G). These results indicated that OE-Sp1 can facilitate BMSC autophagy. Additionally, compared with BMSCs-OE-NC, BMSCs-OE-Sp1 displayed reduced proliferation in CCK8 assays (Fig. 4H), and a significantly increased level of apoptosis, as demonstrated by flow cytometry (Fig. 4I). In conclusion, OE-Sp1 promoted autophagy and impaired the viability of BMSCs under H/SD.

### OE-Sp1 reverses the inhibitory effect of OE-FNDC5 on BMSC autophagy

**Fig. 5:**
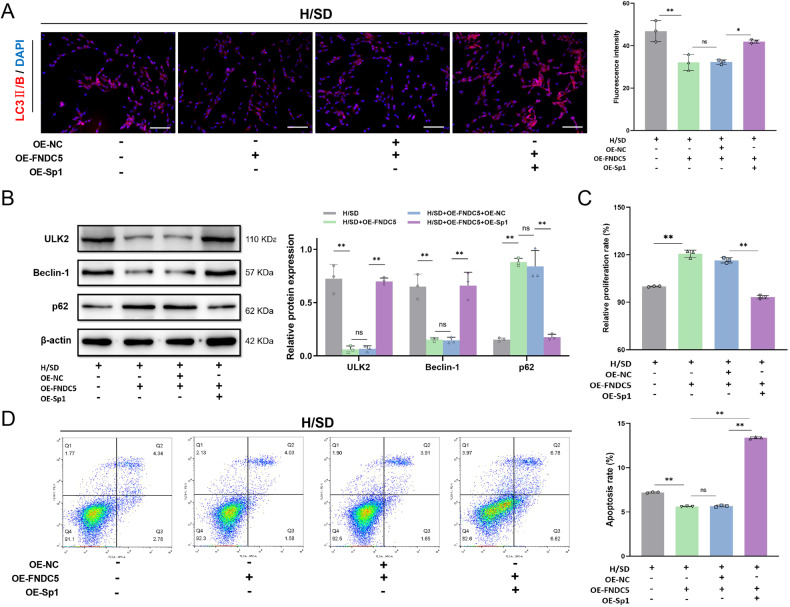
OE-Sp1 reverses the effect of FNDC5 downregulation on autophagy of BMSCs subject to H/SD. **A** Immunofluorescence of LC3 II/B in BMSCs subject to H/SD after OE-FNDC5 lentivirus transfection or OE-Sp1 lentivirus transfection. **B** Western blotting of ULK2, Beclin 1, and p62 proteins in BMSCs. **C** CCK-8 assay detection of cell proliferation after OE-FNDC5 lentivirus transfection or combined with OE-Sp1 lentivirus transfection. **D** Flow cytometry detection of BMSC apoptosis after OE-FNDC5 treatment or combined with OE-Sp1 treatment. Data are presented as the mean ± SD, *p < 0.05, **p < 0.01, *n* = 3, Scale bar = 100 μm.

To confirm that Sp1 plays a key role in FNDC5 regulation of BMSC autophagy, we simultaneously overexpressed Sp1 in BMSCs-OE-FNDC5. LC3 II/B immunofluorescence staining showed that OE-FNDC5 can alleviate the enhanced LC3 II/B expression in BMSCs under H/SD. However, OE-Sp1 reversed this effect of FNDC5 on LC3 II/B (Fig. 5A). Compared with the vehicle control group (OE-FNDC5 + OE-NC), inhibition of OE-FNDC5-induced autophagy was diminished by OE-Sp1 (OE-FNDC5 + OE-Sp1), as evidenced by significantly increased protein levels of ULK2 and Beclin 1, and decreased levels of p62 (Fig. 5B). Moreover, the OE-FNDC5 + OE-Sp1 group displayed impaired proliferation (Fig. 5C) and an elevated rate of apoptosis (Fig. 5D) under H/SD compared with the OE-FNDC5 + OE-NC group, indicating that the inhibitory effect of OE-FNDC5 on BMSC autophagy was reversed by OE-Sp1. In BMSCs under H/SD, FNDC5 down-regulates transcription factor Sp1 and its target autophagy gene, *ULK2*, thereby inhibiting autophagy, decreasing apoptosis, and enhancing cell proliferation.

### FNDC5 promotes the survival of transplanted BMSCs and improves neurological deficits in MCAO rats

**Fig. 6:**
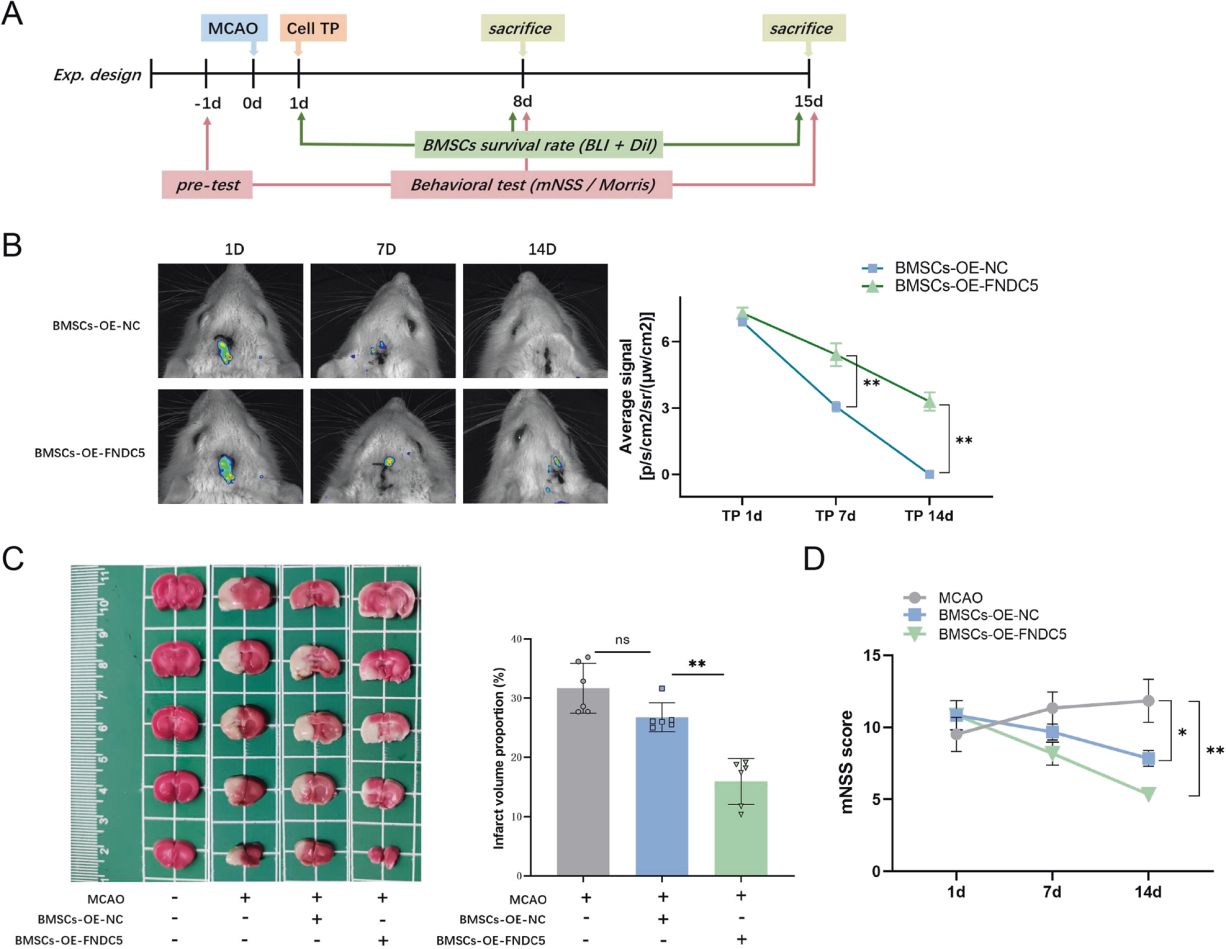
OE-FNDC5 promotes the survival of BMSCs transplanted in a rat cerebral I/R model and improves neurological deficits. **A** Experimental scheme and timeline. **B** Longitudinal bioluminescence imaging spatiotemporally tracked the survival of luciferase-labeled BMSCs-OE-FNDC5 or BMSCs-OE-NC in MCAO rats after cell transplantation at 1, 7, and 14 days. **C** TTC staining evaluated the cerebral infarct volume on day 15 after MCAO. **D** mNSS scores of MCAO rats after BMSC transplantation on days 1, 7, and 14. Data are presented as the mean ± SD, *p < 0.05, **p < 0.01, *n* = 6, Scale bar = 100 μm. MCAO middle cerebral artery occlusion, TP transplantation, BLI bioluminescence imaging, Dil 1,1'-dioctadecyl-3,3,3',3'-tetramethylindocarbocyanineperchlorate staining, mNSS modified neurological severity score.

BMSCs were transplanted into the left lateral ventricle 24 h after MCAO in a stereotactic manner. Behavioral tests were performed prior to and 1, 7, and 14 days after MCAO. To assess the survival of transplanted BMSCs in vivo, luciferase-labeled BMSCs were captured by bioluminescence imaging on days 1, 7, and 14 after BMSC transplantation (Fig. 6A).

Signal gradient variation of bioluminescence indicates the survival rate of BMSCs. Compared with BMSCs-OE-NC, the transplanted BMSCs-OE-FNDC5 displayed substantially stronger fluorescence on days 7 and 14 after cell transplantation, indicating promoted survival of BMSCs overexpressing FNDC5 (Fig. 6B). In addition, cerebral infarct volume on day 14 after cell transplantation was detected by 2,3,4‑triphenyltetrazolium chloride (TTC) staining. Representative TTC-stained brain sections exhibited alleviated ischemic injury (white) in the BMSCs-OE-FNDC5 group compared with the BMSCs-OE-NC group (Fig. 6C) Also, the modified neurological severity score (mNSS) of MCAO rats was improved in the BMSCs-OE-FNDC5-treated group (Fig. 6D). These results showed that the survival of transplanted BMSCs in MCAO rats was promoted by OE-FNDC5. BMSCs-OE-FNDC5 transplantation can significantly improve neurological deficits and alleviate ischemic brain injury in MCAO rats.

### Reversal of promoted survival and enhanced neuroprotective effect of transplanted BMSCs-OE-FNDC5 by OE-Sp1

**Fig. 7:**
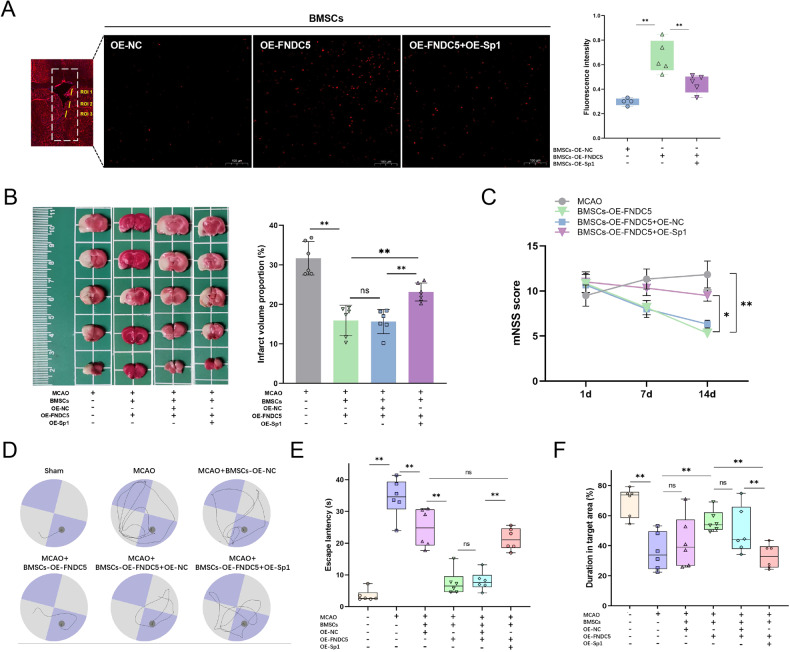
Promoted survival rate and enhanced neuroprotective effect of transplanted BMSCs-OE-FNDC5 are reversed by OE-Sp1. **A** Dil staining tracked the survival of BMSCs-OE-FNDC5 and BMSCs-OE-FNDC5 + OE-Sp1 in the subventricular zone adjacent to the striatum. **B** TTC staining evaluated the cerebral infarct volume 15 days after MCAO. **C** mNSS scores of MCAO rats after BMSC transplantation (on days 1, 7, and 14). **D** Representative swimming paths of the different MCAO and BMSC transplantation groups on day 15. **E** Escape latency of the various MCAO and BMSC transplantation groups. **F** Time spent in the target area by the different MCAO and BMSC transplantation groups. Data are presented as the mean ± SD, *p < 0.05, **p < 0.01, *n* = 6, Scale bar = 100 μm. ROI region of interest.

Survival of BMSCs was also marked by 1,1'-dioctadecyl-3,3,3',3'-tetramethylindocarbocyanineperchlorate (Dil)-staining and traced in the subventricular zone adjacent to the striatum on day 14 after cell transplantation (Fig. 7A). Strong fluorescence signals were recorded in the BMSCs-OE-FNDC5-treated group. However, the BMSCs-OE-FNDC5 + OE-Sp1 group exhibited dramatically decreased fluorescence signals, indicating that the enhanced survival of BMSCs by OE-FNDC5 was diminished by OE-Sp1.

TTC staining showed that treatment of MCAO rats with BMSCs-OE-FNDC5 reduced the ischemic volume. But overexpression of Sp1 in BMSCs reversed the protective effect of BMSCs-OE-FNDC5 (Fig. 7B). Similarly, improvement in the neurological deficits of MCAO rats after BMSCs-OE-FNDC5 treatment was abolished by overexpression of Sp1 (Fig. 7C). Cognitive impairment in MCAO rats on day 14 after cell transplantation was assessed by Morris water maze tests. Compared with the BMSCs-OE-NC treated group, BMSCs-OE-FNDC5 treatment significantly ameliorated learning and memory deficits in MCAO rats, as demonstrated by simplified swimming paths (Fig. 7D). However, MCAO rats in the BMSCs-OE-FNDC5 + OE-Sp1-treated group took much longer to reach the platform and exhibited haphazard swimming paths. In addition, BMSCs-OE-FNDC5-treated MCAO rats showed decreased escape latency (Fig. 7E) and prolonged duration in the target area (Fig. 7F) but these effects were reversed by OE-Sp1. Overall, the protective effect of transplanted BMSCs-OE-FNDC5 on MCAO rats was partially mediated by down-regulation of Sp1.

### BMSCs-OE-FNDC5 transplantation therapy promotes neurovascular proliferation in MCAO rats

**Fig. 8:**
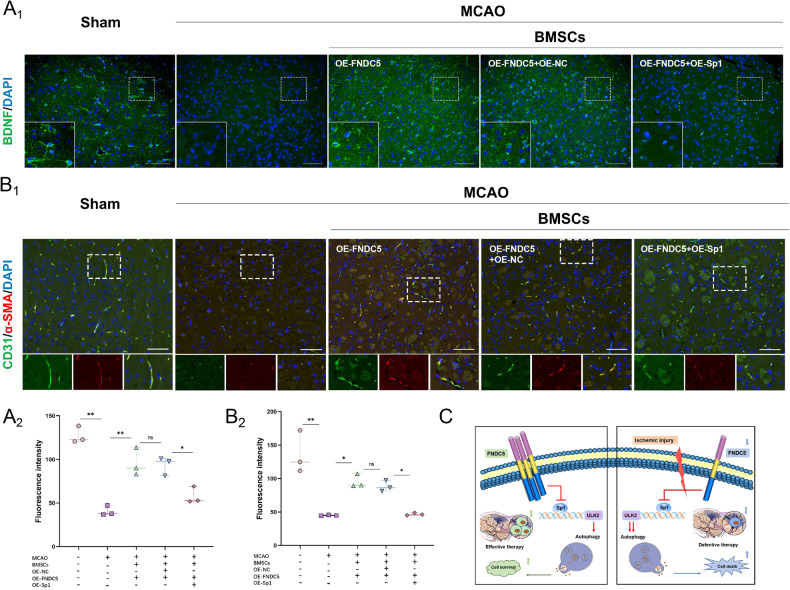
Transplanted BMSCs-OE-FNDC5 promote neurovascular proliferation in MCAO rats, which is reversed by OE-Sp1. **A** Immunofluorescence of BDNF at the edge of the infarct area on day 14 after BMSCs-OE-FNDC5 or BMSCs-OE-FNDC5 + OE-Sp1 transplantation. **B** Immunofluorescence of CD31 and α-SMA at the edge of the infarct area on day 14 after BMSC transplantation. **C** Schematic diagram of the protective effect of FNDC5 on BMSC survival after ischemic injury by regulation of autophagy. FNDC5 down-regulates the transcription factor, Sp1, and reduces expression of the autophagy target protein, ULK2, thereby inhibiting BMSC autophagy, decreasing apoptosis and enhancing cell vitality under hypoxia and nutrient-deprivation. BMSCs-OE-FNDC5 transplantation has a neuroprotective effect in MCAO rats. Data are presented as the mean ± SD, *p < 0.05, **p < 0.01, *n* = 6, Scale bar = 100 μm. BDNF Brain-derived neurotrophic factor, CD31 Platelet endothelial cell adhesion molecule 1, α-SMA α-Smooth muscle actin.

Immunofluorescence staining for the neurogenesis marker, brain-derived neurotrophic factor (BDNF), was performed to detect neurogenesis at the edge of the infarct area on day 14 after cell transplantation. The BMSCs-OE-FNDC5-treated group exhibited prominent expression of BDNF (Fig. 8A). Co-staining for the endothelial marker, platelet endothelial cell adhesion molecule 1 (CD31), and the myofibroblast marker, α-smooth muscle actin (α-SMA) indicated angiogenesis at the edge of the infarct area. BMSCs-OE-FNDC5 treatment also increased vascular density at the ischemic penumbra (Fig. 8B). However, the promotion of neurovascular proliferation by BMSCs-OE-FNDC5 was reversed by overexpression of Sp1.

## Discussion

For treatments to be effective for cerebral infarction they need to restore neurological function [6]. Regenerative medicine, based on MSCs differentiating into substitute neurons in injured brain tissue, provides new opportunities for the development of safe and effective treatments for cerebral infarction [5–8]. In this study, we demonstrated that FNDC5, which is associated with energy metabolism, is a new target for modulating BMSC autophagy. FNDC5 can down-regulate the transcription factor, Sp1, and reduce levels of the autophagy target protein, ULK2, thereby inhibiting BMSC autophagy, reducing apoptosis, and enhancing cell viability in a hypoxic and nutrient-deprived environment. BMSCs-OE-FNDC5 transplantation therapy can significantly alleviate ischemic brain injury, promote neurovascular proliferation, and improve neurological ability in MCAO rats; effects that were mostly attributed to the down-regulation of Sp1 in BMSCs (Fig. 8C).

Adaptive autophagy plays a vital role in maintaining cell homeostasis and energy metabolism [14, 15]. However, under conditions of severe ischemia or prolonged H/SD, destructive autophagy can worsen cellular injury and promote cell death. The hypoglycemic agent, Sitapliptin, promotes the survival of BMSCs in anoxic microenvironments by inhibiting their autophagic activity [24]. The AMPK inhibitor, Compound C, inhibits BMSC autophagy and reduces apoptosis after 24 h of hypoxia [16]. Decreased expression of *ATG7* increases the ability of MSCs to withstand oxidative stress and improves their survival [17]. In our study, autophagy and apoptosis were enhanced in BMSCs subjected to extended H/SD, while the proliferation of BMSCs was decreased. Correspondingly, inhibition of BMSC autophagy can decrease apoptosis and restore the viability of cells under H/SD for 24 h. These results are consistent with those of previous studies [16, 17, 24] and confirmed that reducing destructive autophagy in harsh hypoxic and ischemic environments is conducive to promoting survival of transplanted MSCs. However, more precise genetic, biochemical, and pharmacological studies are needed to fully elucidate the involvement of autophagy in regulating the survival of BMSCs [18]. How can initiation of destructive autophagy be identified? Can destructive autophagy be attributed to hypoxia or nutrient deprivation alone? These questions also warrant further investigation.

FNDC5 and its secreted extracellular cleavage product, Irisin, are widely involved in metabolic processes [25, 26]. Recently, FNDC5/Irisin has been shown to play an important role in modulating autophagy in various disease models [27–29]. In the central nervous system, exercise-induced FNDC5/Irisin release can regulate neuronal autophagy, which is important in anti-aging and synaptic reconstruction [20, 30]. We previously showed that OE-FNDC5 can ameliorate autophagy in BMSCs and promote cell viability upon H/SD [23]. To explore the potential mechanisms for this effect, we performed high-throughput RNA-seq of BMSCs transfected with lentivirus overexpressing FNDC5 (BMSCs-OE-FNDC5) or vector control (BMSCs-OE-NC) that had been subjected to H/SD. Assisted by GO/KEGG cluster analysis, we found that expression of autophagy-related genes in BMSCs-OE-FNDC5 was significantly decreased. Furthermore, the most significant autophagy-related differentially expressed genes were validated by in vitro experiments. qPCR and western blotting confirmed that OE-FNDC5 inhibited the expression of the autophagy trigger, ULK2, in BMSCs. These results further confirmed the protective effect of FNDC5 on BMSCs and revealed molecular targets of autophagy regulation by FNDC5 in BMSCs.

Autophagy is crucial for maintaining cell homeostasis; therefore, we explored further the effect of FNDC5 on autophagy in BMSCs under normal culture conditions. Immunofluorescence showed that similar to the OE-NC group, OE-FNDC5 failed to decrease the level of LC3 II/B in BMSCs under normoxia and sufficient nutrient conditions. Cell viability was approximately equal among groups (Fig. S1). These results indicate that OE-FNDC5 only has a minor effect on BMSC autophagy under normal culture conditions. The heterogeneity of autophagy regulation by FNDC5 can be attributed to diverse autophagy activation ways in varied microenvironments. Cell autophagy is maintained at a normal level when amino acids, ATP and glucose are sufficiently abundant but autophagosome formation is induced upon nutrient depletion. FNDC5 action requires the enzymatic cleavage of a 112 amino acid extracellular segment to form the cytokine, Irisin. Therefore, the varied physiological and pathological function of FNDC5 can be related to the activity of different proteases in the complex cellular microenvironment.

Mammalian Unc-51-like kinases 1 and 2 (ULK1 and ULK2) belong to the Atg1/ULK family of serine/threonine kinases, which are conserved among eukaryotes [31, 32]. Activated AMPK kinase inhibits mTOR phosphorylation, activates the ULK kinase complex and further initiates the autophagy cascade [33, 34]. Therefore, regulation of the Atg1/ULK complex is the switch for overall autophagy regulation. Despite the similarity in their enzymatic domain, ULK1 and ULK2 have substantially different autophagy‑related interactors and post‑translational and transcriptional regulators [35]. Furthermore, ULK1 and ULK2 interactors all have different tissue‑specific expression, which contributes to diverse and ULK‑specific interaction networks in various tissues [35]. Our RNA-seq data analysis showed that OE-FNDC5 inhibited the expression of *ULK2* and not *ULK1* in BMSCs and we verified this by qPCR (Fig. S2). Moreover, we demonstrated that OE-FNDC5 significantly reduced the level of ULK2 and that the transcription factor, Sp1, plays a key role in the regulation of ULK2 by FNDC5. Luciferase assays confirmed the direct effect of Sp1 on ULK2 transcription. These results are consistent with the work of Demeter et al. [35], which indicated that ULK1 and ULK2 have specific transcriptional regulators that are also involved in different processes.

Specificity proteins (Sp) are a well-known family of transcription factors that includes Sp1 to Sp4 [36–38]. These factors are essential for cell growth, differentiation, apoptosis, and carcinogenesis and are implicated in a wide range of crucial processes [37, 39]. Many genes with putative GC-rich Sp-binding sites in their promoters are activated by Sp1 during transcription. In our study, the *ULK2* promoter sequence acquired from the UCSC database was rich in GC-box consensus sequence (see Supplementary 4), and was verified as an Sp1 binding site in the pcDNA-Sp1 transfection luciferase assay. Furthermore, in vitro experiments revealed that OE-FNDC5 down-regulated Sp1 in BMSCs under H/SD. Accordingly, overexpression of Sp1 aggravated the destructive autophagy in BMSCs and reversed the protective effect on cell viability of FNDC5 in BMSCs under H/SD. These results are consistent with previous findings that Sp1 promotes autophagy [40–42]. Furthermore, we have explored ULK2 as a new autophagy target protein of Sp1 and confirmed a key role of Sp1 in the regulation of autophagy by FNDC5 in BMSCs. However, we have not identified the particular pathway through which FNDC5 downregulates Sp1. Post-translational modifications and interactions with other protein factors modulate the activity of Sp1 [37, 43, 44] and FNDC5/Irisin can activate the intracellular kinase signaling pathway through integrin αV/β1 or αV/β5 receptors in the cell membrane [45, 46]. We therefore hypothesize that FNDC5 may modulate various phosphatases in BMSCs and thereby affect the activity of Sp1. We will investigate this hypothesis in more detail in future studies.

Numerous methods have successfully promoted the survival of transplanted stem cells. However, drug trials need to consider toxicity, side effects, and interspecies variation [47, 48]. There are ethical concerns with the clinical application of gene editing technology [49, 50]. Biomedical engineering technology is extremely complex, expensive and difficult to implement widely in a short period of time [51]. Therefore, the development of treatment methods that closely resemble physiology can better advance the clinical translation of MSC transplantation therapy. FNDC5/Irisin is recognized as a myokine that is released into the bloodstream after exercise-induced skeletal muscle contraction [52]. The effect of physical exercise on neurological recovery in MCAO rats treated with stem cells has recently been demonstrated [53, 54]. In the present study, the survival rate of OE-FNDC5-modified BMSCs in MCAO rats was higher than that in the control group. The transplanted BMSCs-OE-FNDC5 alleviated neuronal apoptosis, reduced the cerebral infarct volume, improved neurological defects and promoted neurovascular proliferation in MCAO rats through inhibition of Sp1. These results enrich our knowledge of the molecular mechanisms by which physical exercise enhances the efficacy of MSC therapy. They also promote the clinical application of MSC therapy assisted by physical exercise.

Taken together, our results indicate that FNDC5 down-regulates Sp1 and reduces the expression of *ULK2*, thereby inhibiting BMSCs autophagy, decreasing apoptosis and protecting cell viability upon H/SD. BMSCs-OE-FNDC5 transplantation can significantly alleviate ischemic brain injury, promote neurovascular proliferation, and restore neurological function in MCAO rats. These findings help to reveal novel molecular mechanisms related to MSC-based therapies in cerebral infarction. Preconditioning MSCs with FNDC5/Irisin may shed light on improving the regenerative capacity of MSCs in various ischemic tissues.

## Material and methods

### BMSC preparation and lentivirus transfection

Rat BMSCs were obtained from femurs and tibias of 2-month-old male Sprague–Dawley (SD) rats. After mechanical dissociation of the bone, the marrow was extracted and flushed repeatedly with Dulbecco’s modified Eagle’s medium (DMEM; Invitrogen, Baseley, UK). The collected cell suspension was filtered, centrifuged, and resuspended in DMEM containing 20% fetal bovine serum (FBS; Gibco, Grand Island, NY), 100 U/ml penicillin and 100 mg/ml streptomycin (Sigma-Aldrich, St Louis, MO). These primary cultures of BMSCs were seeded in culture flasks (1×10^8^ cells/flask) and incubated in a 37 °C, 5% CO_2_ incubator. After 24 h the medium was replaced with fresh medium to remove non-adherent cells. The cells were passaged three times and then assessed by flow cytometry before use in experiments.

Once BMSCs reached 50–70% confluency, they were transduced with FNDC5 (Rat FNDC5 Gene ID: 260327), Sp1 (Rat Sp1 Gene ID: 24790), or blank lentivirus for 72 h. Briefly, medium was replaced with fresh medium containing 6 μg/ml polybrene and viral suspension (multiplicity of infection = 10). After 4 h, the polybrene was diluted by adding 2 ml fresh medium. The next day, the transduction medium was replaced with a fresh culture medium. The lentivirus in this study that overexpressed FNDC5 (OE-FNDC5) or Sp1 (OE-Sp1) were commercially provided by Shandong WeiZhen Bioscience Technology. Plasmid vectors were tagged by green fluorescence protein (GFP) or luciferase (for details see Supplementary 5–7). For stem cell tracing, BMSCs were co-cultured with the non-cytotoxic membrane dye, Dil (10 µM; KaiXin, Xi’an, China) for 20 min at 37 °C before transplantation. Transduction efficiency was evaluated under a fluorescence microscope (Olympus, Tokyo, Japan).

### Hypoxia/serum deprivation (H/SD) injury

BMSCs were routinely seeded into 6-well plates (Corning, NY) and cultured in DMEM supplemented with 10% FBS. To mimic hypoxia and ischemia injury in vivo, BMSCs at 70–80% confluency were cultured under hypoxic conditions of 1% O_2_, 94% nitrogen (N_2_), and 5% CO_2_ in glucose-free and serum-free medium for indicated times (12, 24, and 48 h). Control BMSCs were not exposed to H/SD.

### Cell viability assessment

BMSCs were washed with PBS, processed using the Annexin V-FITC Apoptosis Detection Kit (Beyotime, Shanghai, China) according to the manufacturer’s instructions and then detected using a flow cytometer (BD Biosciences, USA). The CCK8 assay was used to evaluate BMSC proliferation under different treatments. In brief, cells were suspended and seeded into 96-well plates (Corning) and cultured under H/SD conditions for 12, 24, and 48 h. CCK8 solution (Beyotime) was added to the medium according to the manufacturer’s instructions and absorbance was measured at 450 nm.

### Luciferase assays

A wild-type plasmid containing a full-length ULK2 promoter sequence (−2000 to 0) and a mutant (MUT) plasmid were constructed and designated pGL4-ULK2-wt and pGL4-ULK2-mut, respectively (for details of the mutant sequence, see Supplementary 8). 293 T cells were co-transfected with either of the above plasmids, a dual Luciferase reporter plasmid and pcDNA3.1-Sp1 or pcDNA3.1-basic. Forty-eight hours after transfection, cells were lysed and centrifuged at 12,000 × *g* for 1 min. The supernatant was collected and luciferase activity measured using the Dual-Luciferase® Reporter Assay System (Dual-Luciferase® Reporter Assay System, E1910, Promega, Madison, WI, USA). For relative luciferase activity calculations, the firefly value was normalized to the *Renilla* value.

### Cerebral ischemia/reperfusion (I/R) rat model

Adult male SD rats (weighing 280–300 g) supplied by the Guangdong Medical Laboratory Animal Center were used to establish the cerebral I/R model. All experimental procedures in this study complied with the guidelines of the Institutional Animal Committee of the Affiliated Yan’An Hospital of Kunming Medical University (Approval ID: 2021035). All rats were maintained on a 12 h light/dark schedule and had free access to chow and water *ad libitum*, unless otherwise specified.

We simulated transient cerebral I/R using the previously described MCAO model [55]. Thirty-six male SD rats were randomly divided into six groups (*n* = 6 rats per group): Sham-control, MCAO, MCAO + BMSCs-OE-NC, MCAO + BMSCs-OE-FNDC5, MCAO + BMSCs-OE-FNDC5 + OE-NC and MCAO + BMSCs-OE-FNDC5 + OE-Sp1. Rats were anesthetized with 10% sodium pentobarbital (1 ml/300 g) by intraperitoneal injection. The internal and external carotid arteries were exposed and isolated and the distal part of the external carotid artery was dissected. Monofilament nylon (4–0) was advanced through the right internal carotid artery to the origin of the middle cerebral artery. I/R was caused by withdrawing the wire after 2 h of embolization. During the surgery, the room temperature was maintained at 22 °C. On completion of all experiments, rats were sacrificed by cervical dislocation. We ensured that animals were handled with the utmost care and all efforts were taken to minimize their suffering.

### Intracerebral transplantation of BMSCs

BMSCs (1 × 10^6^ cells/10 μL DMEM) were injected by intra-cerebroventricular injection (1 μl/min) using a stereotaxic apparatus (68005; RWD, Shenzhen, China) 24 h after MCAO. Stereotactic coordinates were: anteroposterior, 1.1 mm; mediolateral, 1.5 mm; depth, 4.5 mm. The neurological deficit score of MCAO rats was assessed on days 1, 7, and 14 after cell transplantation. Subsequently, rats were sacrificed by decapitation and brain tissues were collected.

### Bioluminescence imaging

Cell tracing by bioluminescence imaging was performed on days 1, 7, and 14 after BMSC transplantation using an AniView100 imaging System (BLT, Guangzhou, China). Briefly, rats were anesthetized and injected intraperitoneally with D-luciferin substrate at 150 mg/kg. They were then placed onto a stage inside the camera chamber, with continuous exposure to isoflurane. Bioluminescence images were acquired 10 min after luciferin injection. Signal intensities from each region of interest (ROI) were quantified as average photons/s/cm2/sr/ (μw/cm^2^) using Living Image Analysis software (BLT AniView100, Guangzhou, China).

### Infarct volume measurement

Seven and fourteen days after BMSC transplantation, the cerebral infarct volume was measured by TTC staining. Briefly, fresh 2-mm-thick coronal brain slices were incubated in 2% TTC (Sigma) at 37 °C for 20 min. The white infarct area was measured using ImageJ software (National Institutes of Health, Bethesda, MD, USA). Cerebral infarct proportion was calculated according to the formula: corrected percentage of infarct volume = total infarct volume/volume of the contralateral hemisphere × 100%. All assessments were conducted under blinded conditions.

### Behavioral tests

#### Modified neurological severity score (mNSS)

Neurological deficits were graded according to the mNSS at 1, 7, and 14 days after MCAO. The mNSS score ranges from 0 to 18 (normal score, 0; maximal deficit score, 18) and consists of various levels of neurological dysfunction to motor, sensory, balance and reflex systems [56]. All assessments were conducted under blinded conditions.

### Morris water maze

The spatial learning and memory of rats were assessed by the Morris water maze test [57]. For four consecutive days, rats received navigation training five times per day. Each training interval was longer than 30 min. Rats were placed into the water of different quadrants facing the wall and the time from entering water to climbing onto the platform was recorded as the escape latency. If a rat could not find the platform within 2 min, they were pulled onto the platform by the investigator and the time was recorded as 120 s. On day 5, the platform was removed. The time spent swimming in the quadrant where the platform had been located was recorded as duration in the target area. The swimming paths, escape latency and duration in the target area were automatically recorded and analyzed by the MWM101 Behavior Analysis System (Bio-will Co., Ltd. Shanghai, China). All assessments were conducted under blinded conditions.

### Immunofluorescence staining

The autophagy representative marker, Microtubule-associated protein one light chain 3 II/B (MAP1LC3/LC3 II/B) was detected by immunofluorescence. BMSCs in 6-well plates were fixed by incubation with 4% paraformaldehyde at room temperature for 15 min. After rinsing 3 × 15 min in PBS, the wells were incubated with blocking solution, followed by incubation with the primary LC3 II/B antibody (1:1000, Abcam, Cambridge, UK) for 1 h at room temperature. After washing three times with PBS, cells were incubated with an anti-mouse FITC-conjugated secondary goat antibody (1:500, Servicebio, Wuhan, China) in a blocking solution for 45 min at room temperature shielded from light. Before imaging, the wells were incubated with DAPI (1:500, Servicebio, Wuhan, China) for nuclear staining. Images were acquired using an upright fluorescence microscope (Olympus).

To detect neuron survival and endogenous neurovascular proliferation at the edge of the infarct area, immunofluorescence staining of BDNF, CD31, and α-SMA was performed. Briefly, six rats were randomly selected for each group. Brain sections were incubated in 0.2% Triton X-100 (Beyotime) for 10 min and blocked with 10% goat serum in PBS for 30 min at room temperature. Then, sections were incubated overnight at 4 °C with primary antibodies against BDNF (1:100, ABclonal Technology, Wuhan, China), CD31 (1:100, ABclonal Technology), and α-SMA (1:200, Proteintech, Wuhan, China). After rinsing 3 × 15 min in PBS, the sections were incubated with a biotinylated anti-rat secondary antibody (1:500, ABclonal Technology) and DAPI for 60 min at room temperature. Finally, coverslipped slides were photographed under a BX53 microscope (Olympus).

### Quantitative real‑time PCR (qRT‑PCR)

Total RNA was isolated from cells using Trizol reagent (Invitrogen, Carlsbad, CA, USA) following the manufacturer’s instructions. RNA preparations were treated with PrimeScript Reverse Transcriptase (Takara, Dalian, China) to generate cDNAs. qRT-PCR was performed using DNA Master SYBR Green I mix (Qiagen, Hilden, Germany) on a StepOnePlus^TM^ system (Applied Biosystems, Carlsbad, CA, USA). Data were normalized against β-actin and relative gene expression was calculated using the 2^−ΔΔCT^ method. ΔCt = (Ct of gene of interest − Ct of actin). The qRT-PCR primers were designed using Primer3Plus software and are listed in Table 1.

**Table 1:** Primers used in real-time polymerase chain reaction.

Gene	Sequence (5' → 3')	Produce size (bp)
R-ATG12-F	CGGACTGTCCAAGCACTCAT	112
R-ATG12-R	CTTCTTGGTCTGGGGAAGGG	
R-ATG4C-F	GCTCGTGACAAGGCTGTTAT	128
R-ATG4C-R	TTGCCGCCAATGATACCAAC	
R-ULK1-F	ATGGTACAATCAGCTGCACTCG	125
R-ULK1-R	TCAATGTCTGCCTGGTCCGT	
R-ULK2-F	TGGAGCATAGGAACGGTGAT	167
R-ULK2-R	TGAAGCAAGCCCAAAAGGAG	
R-Sp1-F	CAGCAGCGGATCATCAGGG	121
R-Sp1-R	TCCTTGCAGTGATCCTCCT	
R-FNDC5-F	TAACCGTCAGGCACCTCAAG	115
R-FNDC5-R	CGCAGCATCCTCACATCCTT	

### Western blotting

Collected cells were lysed in radioimmunoprecipitation assay buffer (Beyotime) for protein extraction. Protein concentration was measured using a Bradford assay kit (Beyotime). Protein samples were separated by 12% sodium dodecyl sulfate‐polyacrylamide gel electrophoresis and transferred to polyvinylidene fluoride membranes (Millipore, USA). After blocking in 5% non-fat milk for 1 h, the membranes were incubated with primary antibodies against Beclin 1, p62, FNDC5, Sp1, ULK2 (1:1000; all from ABclonal) or β-actin (1:2000; Santa Cruz, VA, USA) at 4 °C overnight. After rinsing with TBS-T buffer, membranes were incubated with an appropriate horseradish peroxidase-conjugated secondary antibody (1:5000, ABclonal) for 2 h. Membranes were treated with an enhanced chemiluminescence (ECL) Plus Western Blotting Detection Kit from GE Healthcare and protein bands were then visualized using an ECL detection system (Tanon 4600, Shanghai, China). All values were normalized against an equal loading control.

### RNA sequencing and bioinformatics analysis

RNA-seq was performed on BMSCs-OE-FNDC5 and BMSCs-OE-NC that were cultured under H/SD conditions for 24 h. The sequencing was performed on the Illumina sequencing platform by Novogene. Acquired DEGs were subjected to GO functional and KEGG pathway enrichment analysis. We screened out the three most significantly differentially expressed autophagy-related genes (∣log FC∣ ≥ 1, *p* value < 0.05) for experimental validation. The FASTA sequences (first 2000 bp) of the above three autophagy-related genes were retrieved from the UCSC database (https://genome.ucsc.edu) and used to predict transcription factors in the JASPAR database (https://jaspar.genereg.net). Finally, ten predicted transcription factors with a correlation rate greater than 90% were further intersected with DEGs identified from the RNA-seq data.

### Statistical analysis

All quantitative data are presented as the mean ± standard deviation of n independent experiments. Comparison among multiple groups was analyzed using ANOVA followed by Tukey’s post hoc test. Student’s *t* test (unpaired, two-tailed) was used to analyze differences between two groups. All statistical tests were performed using GraphPad prism 9.0, and *p* value < 0.05 was considered statistically significant. No sample size estimate was performed, but sample size was selected based on previous experiments. All assays were repeatable in independent experiments and the figures presented are representative.

### Supplementary information

Figure S1

Figure S2

Legends for Fig S1-S2

Supplementary 4 ULK2 promoter Seq

Supplementary 5 Plasmid FNDC5-GFP

Supplementary 6 Plasmid FNDC5-LUC

Supplementary 7 Plasmid Sp1-GFP

Supplementary 8 ULK2 TFBS mutation

Full length western blots

## Data Availability

The datasets generated during the current study are available from the corresponding authors upon reasonable request. The RNA-seq data have been deposited in NCBI’s Gene Expression Omnibus (Edgar et al., 2002) and are accessible through GEO Series accession number GSE231837.
