# Supplementary figures and images for: FNDC5 inhibits autophagy of bone marrow mesenchymal stem cells and promotes their survival after transplantation by downregulating Sp1
Source: Cell Death Discov. 2023 Sep 6;9:336. doi: 10.1038/s41420-023-01634-4 (PMC10482879; doi:10.1038/s41420-023-01634-4)

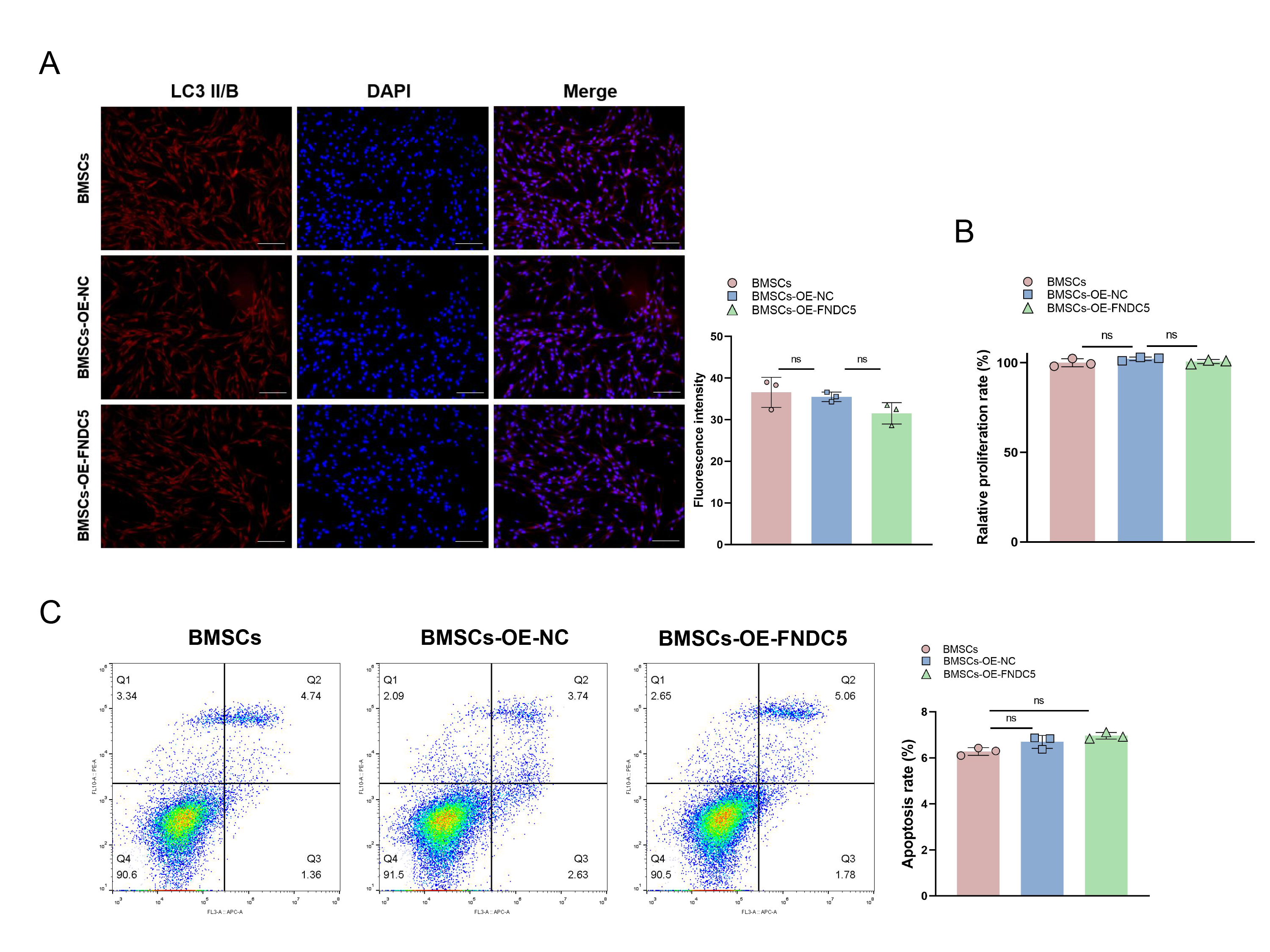

Supplement: Supplementary file 1 — Figure S1 [file 41420_2023_1634_MOESM1_ESM.png]

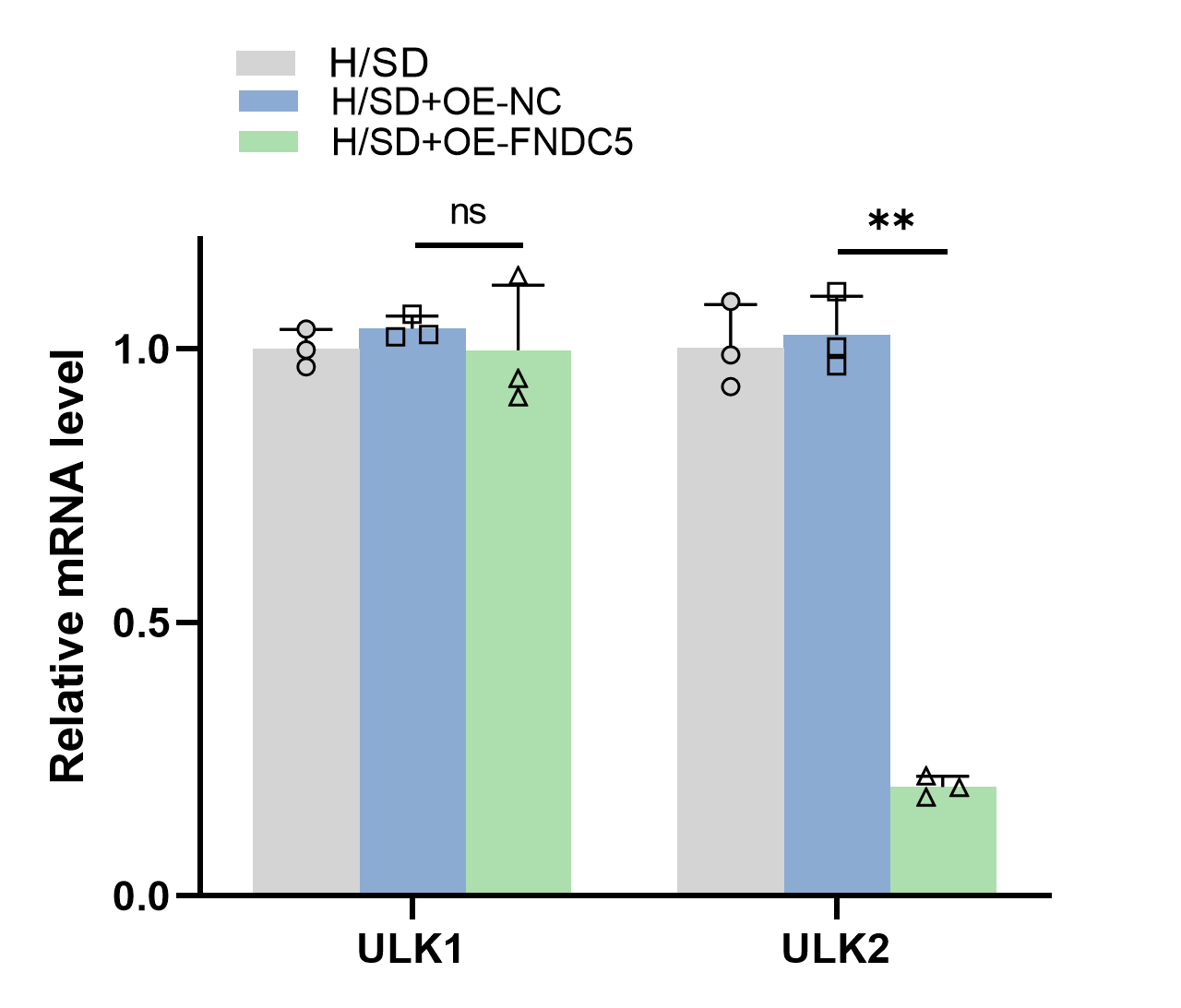

Supplement: Supplementary file 2 — Figure S2 [file 41420_2023_1634_MOESM2_ESM.tif]

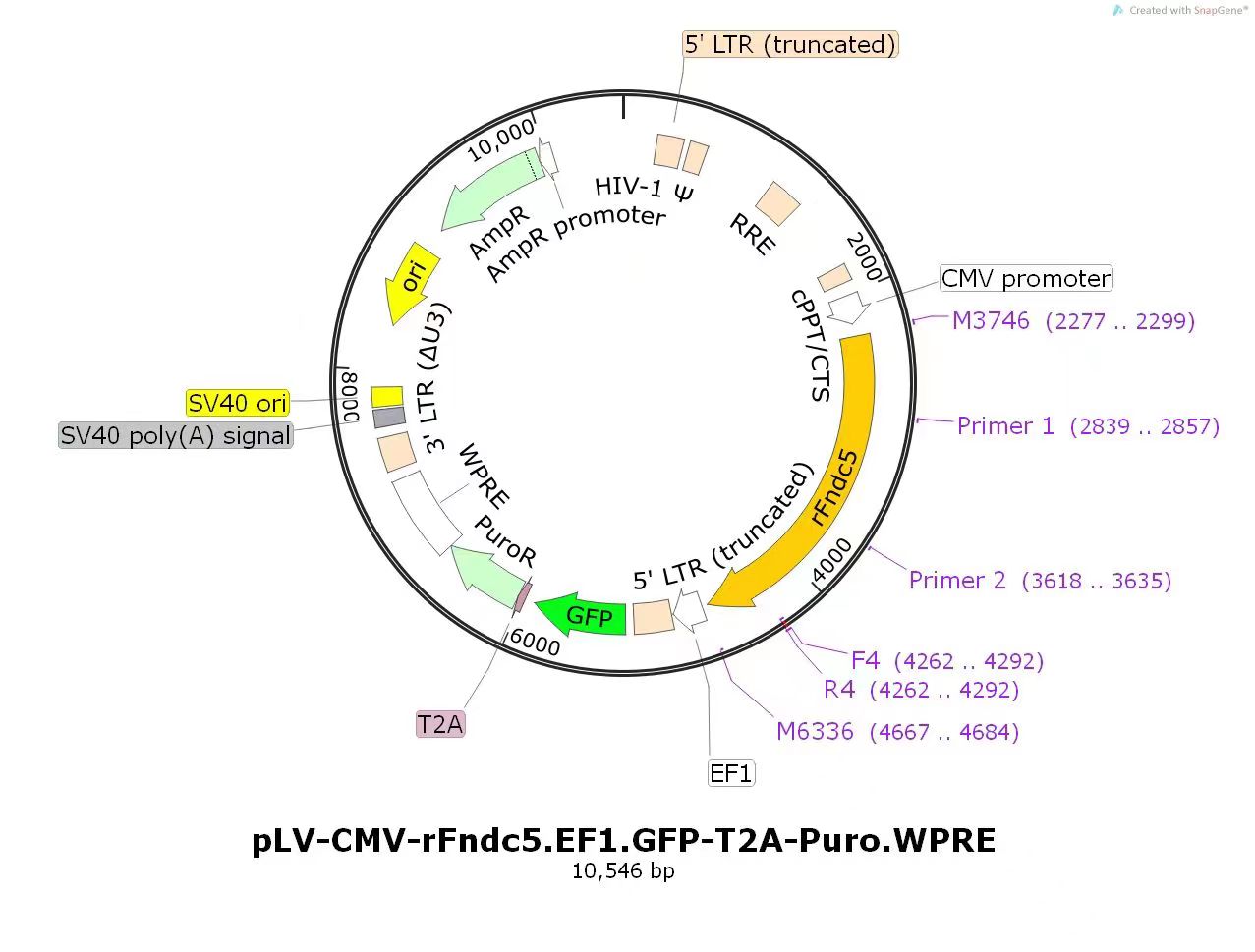

Supplement: Supplementary file 5 — Supplementary 5 Plasmid FNDC5-GFP [file 41420_2023_1634_MOESM5_ESM.jpg]

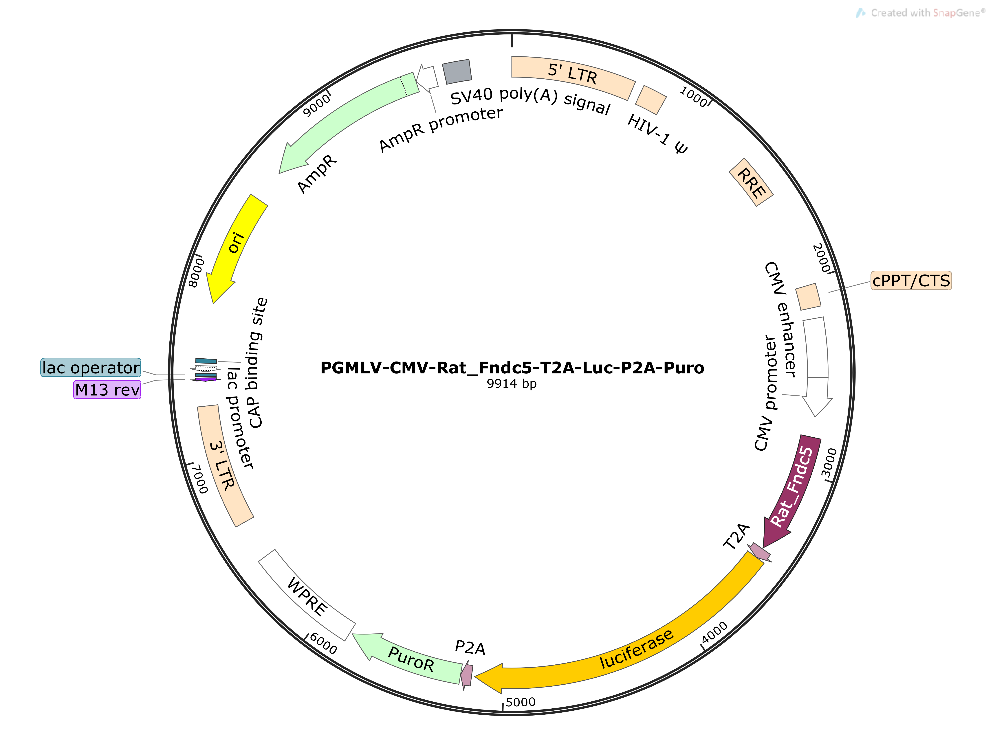

Supplement: Supplementary file 6 — Supplementary 6 Plasmid FNDC5-LUC [file 41420_2023_1634_MOESM6_ESM.png]

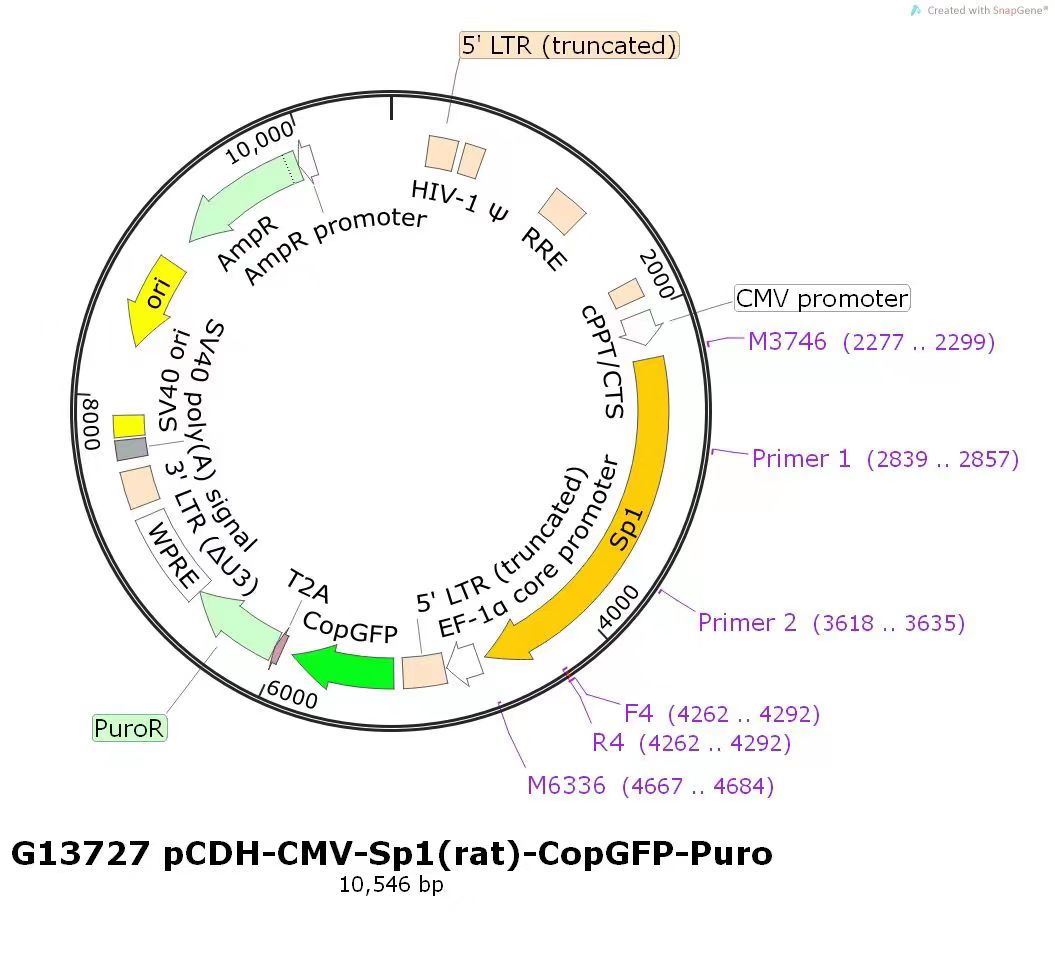

Supplement: Supplementary file 7 — Supplementary 7 Plasmid Sp1-GFP [file 41420_2023_1634_MOESM7_ESM.jpg]

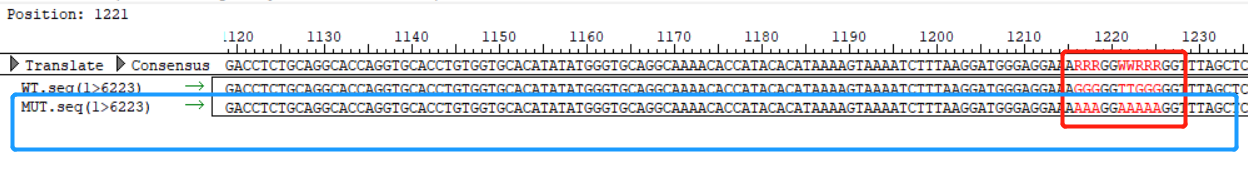

Supplement: Supplementary file 8 — Supplementary 8 ULK2 TFBS mutation [file 41420_2023_1634_MOESM8_ESM.png]
