# Supplementary material for: FNDC5 inhibits autophagy of bone marrow mesenchymal stem cells and promotes their survival after transplantation by downregulating Sp1
Source: Cell Death Discov. 2023 Sep 6;9:336. doi: 10.1038/s41420-023-01634-4 (PMC10482879; doi:10.1038/s41420-023-01634-4)
Supplement: Supplementary file 3 — Legends for Fig S1-S2 [file 41420_2023_1634_MOESM3_ESM.docx]

**Fig. S-1** **OE-FNDC5 has small effects on BMSC autophagy and viability under normal culture conditions.** **A** Immunofluorescence of LC3 II/B in BMSCs cultured under normoxia and nutrient-sufficiency after transfection of OE-FNDC5 lentivirus or vector control. **B** CCK-8 assay detection of BMSC proliferation after OE-FNDC5 lentivirus or vector control transfection under normal culture conditions. **C** Flow cytometry detection of BMSC apoptosis after OE-FNDC5 lentivirus or vector control transfection under normal culture conditions. Data are presented as the mean ± SD, **p* < 0.05, ***p* < 0.01, n = 3, Scale bar = 100 μm.

**Fig. S-2 OE-FNDC5 decreased the mRNA level of *ULK2* not *ULK1*.** qRT-PCR demonstrated that the mRNA level of *ULK2* was significantly decreased by OE-FNDC5 in BMSCs under H/SD. However, OE-FNDC5 had only a minor effect on the mRNA level of *ULK1.* Data are presented as the mean ± SD, ***p* < 0.01, n = 3.
