# Supplementary material for: FNDC5 inhibits autophagy of bone marrow mesenchymal stem cells and promotes their survival after transplantation by downregulating Sp1
Source: Cell Death Discov. 2023 Sep 6;9:336. doi: 10.1038/s41420-023-01634-4 (PMC10482879; doi:10.1038/s41420-023-01634-4)
Supplement: Supplementary file 4 — Supplementary 4 ULK2 promoter Seq [file 41420_2023_1634_MOESM4_ESM.docx]

>ratULK2-promoter

GTTGCCTTCTGGGTGGGTCTGCTATAAAAGTAGCTAGGGAGGGCCTGTGAGATGGTTAGG

ACACAGGTGCTTGTCATGTCACCAAGGCTACCTAACTGAGTTCCACCCCCAGGTCCCATA

TGGTGGAGGGAAAAACCTGACTCCTGCAAGTGATCCTCTGACCTCCACATAAGGGAGTGA

AAGCACACACACAATAAATACATATATTTTACAAGGTGGTTAAGTGTTGGAGCTAGAACT

CAGTGGCAGAGAACTGCTTAGCATGTGTGAAGTCCTAGGTCTGATCCTCAACACCTAGGA

GAGAAAAAAAGGTCCAGCTTGCCTAGGTCCCTCATACTTCCTGGATCTCATGTTAGGATG

CTTTGAGATACCTTGTGCCACTTCAGGACTCTGTCAGCAAGAAAGTTGTCACCAGATACA

ACCCTCTACTGTGGACAAGAACTACAAGTTTAAAAAAAAAAAGAAAAGAAAATCTAATTT

ACTGTTATGATTTGGGGTTTGGATGGTTTGAATGTGTTCCTCAAAGTGCTGGATGCTTGG

ACCTTGGTGTGGTGAGATGTGAATTGAATTGAGAGACCATGACATTGGTTCTTCTGAGAG

TGAGTTGTTATTAAGGAGTAACCCTGGTCCCTTCTCAGTCTCGGGCTTCTCTGCCCTGTG

ATCTCTGTTCCATCATCCCTTGTGAAGCCCTTCTTCATGAGCTCCTTATCAGAGCTGGTG

CTTTGCTGTTTGGGGTGTAAGCTTCCAAATTGTGAGCTACATAAATCTCATTTCTTTACA

AGGTGCCAAGCCTGGGGTGTTTTGTTCTAGCAATAGAAAATGGACTTACCGAGTCTTTTT

TTTTTTTTTTTTTTCCTTCTTCAGGAAAGTGGACCAGAAACATTGCTTTCAGATTCCTGA

CCTGCCTCAAAGCTGGAGGTTTTTAGGACCCCTCCTAGTTGTCAAGATGCGTTTTAGAGC

GCTGGCTGGAGAGACAGCTCAGTGGTTTAAGAGCACTGGCTATTATTCTATCAGAGGACT

TGGGTTTGATTTCTAATACCTACAATTGTGGCTCACAGCCATCTGTAATTCCAGTCTCAG

GAGATCGACACCCCCATCTGACCTCTGCAGGCACCAGGTGCACCTGTGGTGCACATATAT

GGGTGCAGGCAAAACACCATACACATAAAAGTAAAATCTTTAAGGATGGGAGGAAAGGGG

GTTGGGGGTTTAGCTCAGCGGTAGAGCACTTGCCTAGCAAGCACAAGGCCCTGGGTTCGG

TCCCCAGCTCCGAAAAAAAAAGAAGAAAAAAAAAGGATGGGGGGGAAGGAGACACAGACA

GACAGACAGACAGACAGACAGACAGACAGACAGGCGGGCGGGCGGGCGGACGGACGGACG

GACGGACGGACGGACGGACCGACGGGTTTCAGGGGCAGGCAATGAAATTGTGATGAGGTA

ATTAGATAGCAACATCCCCAAACCTGAACACACACTTAGGTGTGGGGTATGTATATTGTT

TACCATGGTTTTGTAATGCTTCTTTGAGCAAAAGTTGACAAATCATTTTTCACCTGAACA

ATTGTAAAAGAGGAGGAAAAAAAAATCTGAGGCAAACTGGTAGTTCACTGGCGTGAAGAA

GGTTGTCGATAGAAAGAAGATTCTAAGCCTCTTTTAGAAGTCTAGAGACCCCCCCAAAAG

GCGCAGGTCCCAGTCACACCTATCCCTTCAGTGGGTTGTCCAGCAGCTTGCGGTCTGGAT

TTGAAAGGCCGCTCGCTGAGTTTTTGTCTTCTATGTCTGCACCCCCTCAGCGATTACCCC

GGCCAGAGTTCAAGATCCGCCCCTTAGAGGGTGGCTTCAGGTCAGGGCGTGGCGGGCTCC

AGCTAACCGCCGGCGCCGAGCCGGCCGCCGGTTCCTAAGCAGCCGTCGCCTCCCCGGCTC

GCCGACTCGCTCCAGTGACTGCAGATTCGCCCCGGTGCCTGGCGCCGCGTCCCGGGGCGC

GCACTCCTTTTCTCGGCGGA
