# Supplementary material for: FNDC5 inhibits autophagy of bone marrow mesenchymal stem cells and promotes their survival after transplantation by downregulating Sp1
Source: Cell Death Discov. 2023 Sep 6;9:336. doi: 10.1038/s41420-023-01634-4 (PMC10482879; doi:10.1038/s41420-023-01634-4)
Supplement: Supplementary file 9 — Full length western blots [file 41420_2023_1634_MOESM9_ESM.docx]

**Original Western Blot images (unprocessed)**

**Fig. 1 C**

Groups (from the left to the right): Control, H/SD 12h, H/SD 24h, H/SD 48h.

1. Beclin-1





1. p62





1. β-actin





**Fig. 2 F**

Groups (from the left to the right): H/SD, H/SD+OE-NC, H/SD+OE-FNDC5.

1. ULK2





1. Beclin-1





1. p62





1. β-actin





**Fig. 3 D**

Groups (from the left to the right): Control, H/SD, H/SD+OE-NC, H/SD+OE-FNDC5.

1. Sp1





1. β-actin





**Fig. 4 B**

Groups (from the left to the right): Control, OE-NC, OE-Sp1.

1. Sp1





1. β-actin





**Fig. 4 E**

Groups (from the left to the right): H/SD, H/SD+OE-NC, H/SD+OE-Sp1.

1. ULK2





1. β-actin





**Fig. 4 G**

Groups (from the left to the right): H/SD, H/SD+OE-NC, H/SD+OE-Sp1.

1. Beclin-1





1. p62





1. β-actin





**Fig. 5 B**

Groups (from the left to the right): H/SD, H/SD+OE-FDNC5, H/SD+OE-FNDC5+OE-NC, H/SD+OE-FNDC5+OE-Sp1.

1. ULK2





1. Beclin-1





1. p62





1. β-actin
